# Study of Adsorption and Flocculation Properties of Natural Clays to Remove *Prorocentrum lima*

**DOI:** 10.3390/toxins7103977

**Published:** 2015-09-29

**Authors:** Maria Carmen Louzao, Paula Abal, Diego A. Fernández, Mercedes R. Vieytes, José Luis Legido, Carmen P. Gómez, Jesus Pais, Luis M. Botana

**Affiliations:** 1 Departamento de Farmacología, Facultad de Veterinaria, Universidad de Santiago de Compostela, Lugo 27002, Spain; E-Mails: paula.abal@usc.es (P.A.); alberto.fernandez@usc.es (D.A.F.); 2 Departamento de Fisiología Animal, Facultad de Veterinaria, Universidad de Santiago de Compostela, Lugo 27002, Spain; E-Mail: mmercedes.rodriguez@usc.es; 3 PeloidesTermales S.L., Fonte das Abelleriras s/n. Edificio CITEXVI, Vigo 36310, Spain; E-Mails: xllegido@uvigo.es (J.L.L.); info@peloides.org (C.P.G.); 4 Caolines de Vimianzo S.A.U., (CAVISA), Cerbán-Castrelo 19, Vimianzo A Coruña 15129, Spain; E-Mail: jesus.pais@e-cavisa.com

**Keywords:** *Prorocentrum lima*, dinoflagellate, clay, adsorption, flocculation

## Abstract

High accumulations of phytoplankton species that produce toxins are referred to as harmful algal blooms (HABs). HABs represent one of the most important sources of contamination in marine environments, as well as a serious threat to public health, fisheries, aquaculture-based industries, and tourism. Therefore, methods effectively controlling HABs with minimal impact on marine ecology are required. Marine dinoflagellates of the genera *Dinophysis* and *Prorocentrum* are representative producers of okadaic acid (OA) and dinophysistoxins responsible for the diarrhetic shellfish poisoning (DSP) which is a human intoxication caused by the consumption of shellfish that bioaccumulate those toxins. In this work we explore the use of natural clay for removing *Prorocentrum lima*. We evaluate the adsorption properties of clays in seawater containing the dinoflagellates. The experimental results confirmed the cell removal through the flocculation of algal and mineral particles leading to the formation of aggregates, which rapidly settle and further entrain cells during their descent. Moreover, the microscopy images of the samples enable one to observe the clays in aggregates of two or more cells where the mineral particles were bound to the outer membranes of the dinoflagellates. Therefore, this preliminary data offers promising results to use these clays for the mitigation of HABs.

## 1. Introduction

Massive algal proliferations known as Harmful Algal Blooms (HABs) are natural phenomena that constitute one of the most important sources of contamination in the oceans [1]. As a result of HABs, due to the frequent presence of toxin-producing organisms, biotoxins are accumulated across food chains causing adverse effects for animals and humans and threatening ecosystem integrity [2]. Anthropogenic activities appear to contribute to the increased incidence of HABs registered in recent years, by the introduction of non-native species through ballast water [3], environmental change caused by eutrophication or pollution load [4], and global climate change [5,6].

Among all microalgae, the genus *Prorocentrum* and *Dinophysis* of marine dinoflagellates synthesize okadaic acid (OA) and dinophysistoxins (DTXs) [7]. Those toxins are accumulated within the tissues of filtering organisms feeding on HABs, rapidly spreading to their predators in the food chain and eventually reaching human consumers causing diarrhetic shellfish poisoning (DSP) syndrome [8]. DSP is a gastrointestinal disease that appears within 30 min to few hours after the ingestion of mollusks that concentrated lipophilic toxins mainly in the digestive glands [9].

OA and DTXs are potent inhibitors of serine/threonine protein phosphatases 1 and 2A (PP1, PP2A) [10,11]. These phosphatases inhibition lead to the phosphorylation of many proteins, which are involved in a large number of processes. All these events can collapse regulatory routes and cause a variety of cellular alterations, resulting in some cases to tumorigenic and embryotoxic effects, cytoskeleton rearrangements, disruption of cell interactions, and even apoptosis induction [12,13,14].

Several strategies have been studied for removing HABs such as the biological and microbial algaecides [15,16,17], chemical [18], mechanical [19], and genetic measures [20]. However, these methods have drawbacks, such as the introduction of new species to control the algae population, the use of agents that could be harmful to other organisms and environmentally damaging or the inapplicability to large scale [21]. So far few technologies can be applied successfully at large scales in natural waters in an ecologically safe and cost-effective way [22]. Therefore, methods effectively controlling HAB with minimal impact on marine ecology are required.

The use of clays for removing microalgae has been previously explored. Their physical and chemical characteristics are mainly responsible for absorption properties [23]. Swelling clay minerals are widespread on the earth’s surface. Swelling clay minerals are widespread on the Earth’s surface. These compounds are interesting due to their large specific surface area and high cation exchange capacity; the latter characteristic provides adsorptive capacity. When dispersed in water, clay minerals present a wide range of colloidal behavior that differs with the chemical composition, size, and shape of the clay and the nature of the exchangeable cation [24]. In this sense, naturally abundant clay minerals such as kaolinite and bentonite can adsorb various biomolecules in the environment. Kaolinite and bentonite are two different phyllosilicate clays. Bentonite is a phyllosilicate type 2:1 with octahedral matrices. Bentonite is composed mainly of montmorillonite, a clay mineral of the smectite group, and is produced by *in situ* devitrification of volcanic ash. The special properties of bentonite are the ability to form thixotrophic gels with water and to absorb large quantities of water and a high cation exchange capacity. Kaolinite is a phyllosilicate type 1:1; it has two-dimensional matrices of tetrahedrons. Kaolinite is made up of tiny sheets of triclinic crystals with pseudohexagonal morphology. Rock weathering forms it. It has also cation exchange capacity. They may have applicability in ecosystem safety due to their potential abilities to remove cells such as cyanobacteria and dinoflagellates from freshwater or seawater [19]. However the studies on this application are still limited. The aim of the present work was to evaluate the adsorption and flocculation properties of different clays in seawater containing *Prorocentrum lima*.

## 2. Results and Discussion

**Figure 1:**
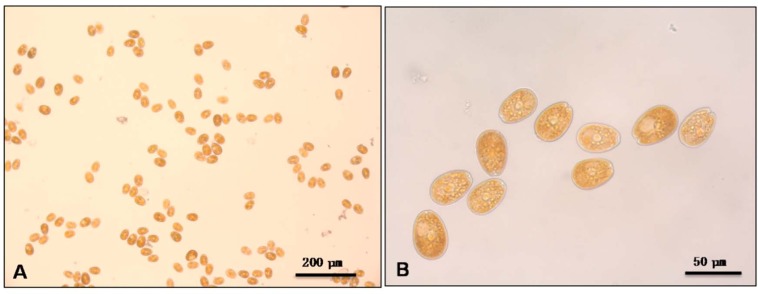
Microscopy images of *Prorocentrum lima* cells; (**A**) 10X; (**B**) 40X.

We choose the microalgae *Prorocentrum lima* because it is one of the representative producers of okadaic acid and dinophysistoxins responsible for the diarrhetic shellfish poisoning (DSP). It is a benthic microalgae that tend to continuous sedimentation, in our experimental conditions, for more than 4 h. *Prorocentrum lima* (Figure 1) was incubated in seawater with 0.2% of different clays and the mixture was allowed to settle by gravity.

**Figure 2:**
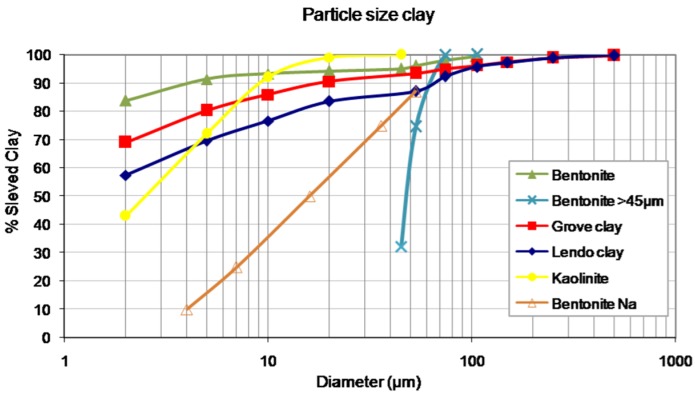
Particle size clay determined from the percentage of sieved clay for each pore diameter: Bentonite (83% < 2 µm), Bentonite > 45 µm (90% > 45 µm), Lendo Clay (57% < 2 µm), Grove Clay (69% < 2 µm), Kaolinite (43% < 2 µm), and Bentonite Na (80% < 70 µm).

The clays were bentonite, sodium bentonite, “Bentonite > 45 µm”, kaolinite, and kaolinites locally available from the regions Lendo and Grove (Figure 2).

**Figure 3:**
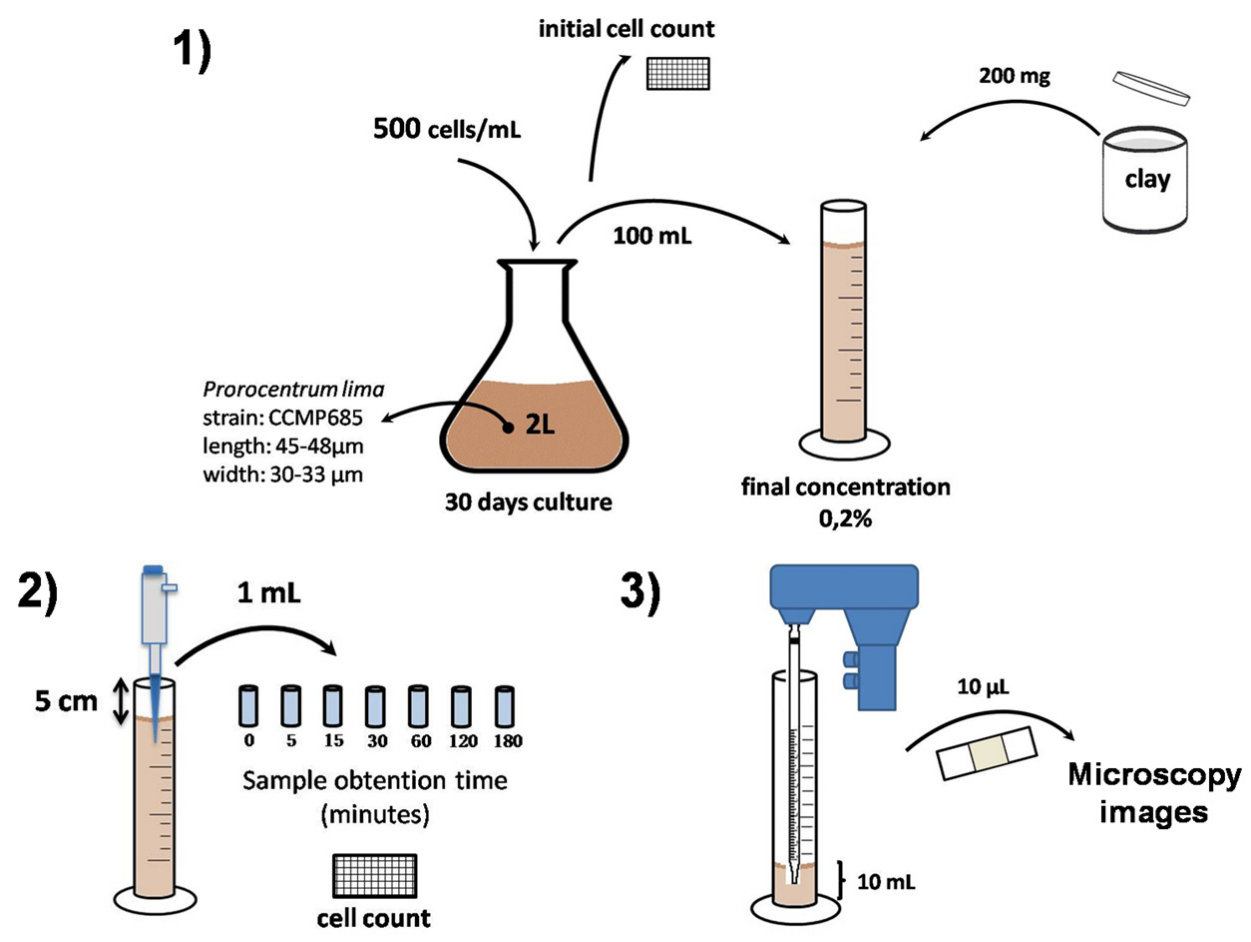
Assay scheme. (**1**) *Prorocentrum lima* culture conditions and preparation of water columns; (**2**) Sampling and cell count; (**3**) Sample preparation for microscopy.

1 mL samples were taken at different incubation times. The reduction of the algal cells was recorded for 180 min (Figure 3).

**Figure 4:**
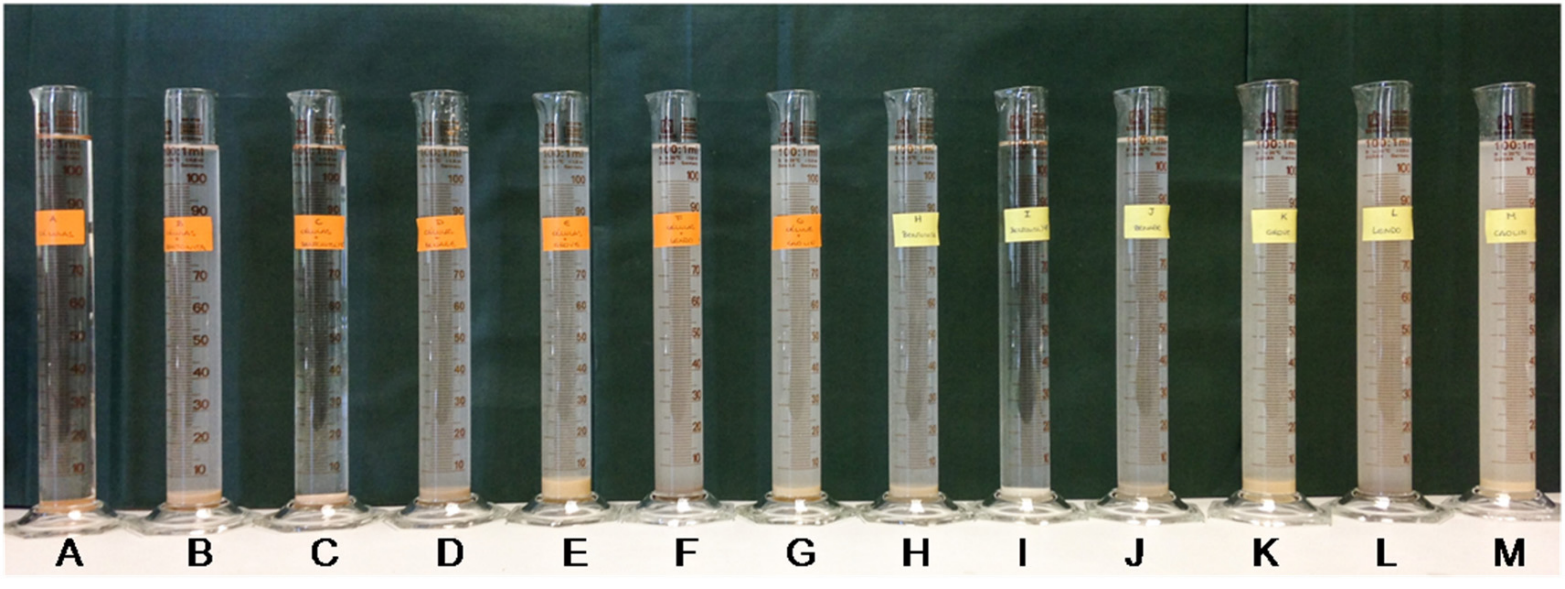
*In vitro* flocculation of clay particles and *Prorocentrum lima* in seawater. Seawater containing different clays was incubated with (orange label) or without (yellow label) *Prorocentrum lima*. Sedimentation of dinoflagellate cells was continuously recorded. (**A**) cells of *Prorocentrum lima*; (**B**,**H**) bentonite; (**C**,**I**) bentonite > 45 µm; (**D**,**J**) bentonite Na; (**E**,**K**) Grove clay; (**F**,**L**) Lendo clay; and (**G**,**M**) kaolinite.

We set up 100 mL water columns with seawater, the corresponding clay (final concentration 0.2%) and the dinoflagellate *Prorocentrum lima* (Figure 4). The mixtures were allowed to settle by gravity. We tested different concentrations of clays (data not shown) and selected 0.2% based on the removal efficiency. Concentrations higher than 0.2% completed decantation fast, after the clay suspension preparation. However, lower clay concentrations were not effective on flocculation of dinoflagellate cells.

All clays were first dispersed in a small volume of seawater before this suspension was added to the 100 mL water columns. The goal of the assay is the cell removal through the flocculation of algal and mineral particles leading to the formation of aggregates, which rapidly settle and further entrain cells during their descent.

The dinoflagellate immediately reacted to the addition of clays, particularly kaolinite, and cells began to precipitate in 5 min. Continuous sedimentation and significant cell removal was almost completed at 30 min (Table 1).

**Table 1:** Cell count (100 µL sample) at the moment of adding clays (0 min) and after 5, 15, 30, 60, 120, and 180 min. * These values were not statistically different from controls of *P. lima*.

Mean Cells ± SEM (*n* = 3)							
Column Content	Time (Minutes)						
	*0*	*5*	*15*	*30*	*60*	*120*	*180*
*P. lima*	631 ± 7.1	447 ± 20.9	267 ± 26.5	149 ± 32.5	91 ± 30.8	56 ± 18	47 ± 14.8
*P. lima* + bentonite > 45 µm	557 ± 29.4	297 ± 4.9	156 ± 42.5 *	74 ± 26.4 *	25 ± 6.6 *	16 ± 3.2	11 ± 2.3
*P. lima* + Lendo clay	521 ± 11.7	287 ± 22.6	81 ± 12	15 ± 8.1	3 ± 2	1 ± 0.3	0
*P. lima* + Grove clay	536 ± 11.9	215 ± 44.2	61 ± 10.9	14 ± 8.8	3 ± 2.3	0	0
*P. lima* + bentonite	557 ± 18.6	221 ± 40.6	49 ± 23.8	9 ± 2.4	2 ± 0.3	0	0
*P. lima* + kaolinite	441 ± 27.4	209 ± 31.3	48 ± 6.5	14 ± 3.8	2 ± 1.5	0	0
*P. lima* + bentonite Na	506 ± 13.8	141 ± 35.7	67 ± 20.3	9 ± 1.2	1 ± 0.3	0	0

**Figure 5:**
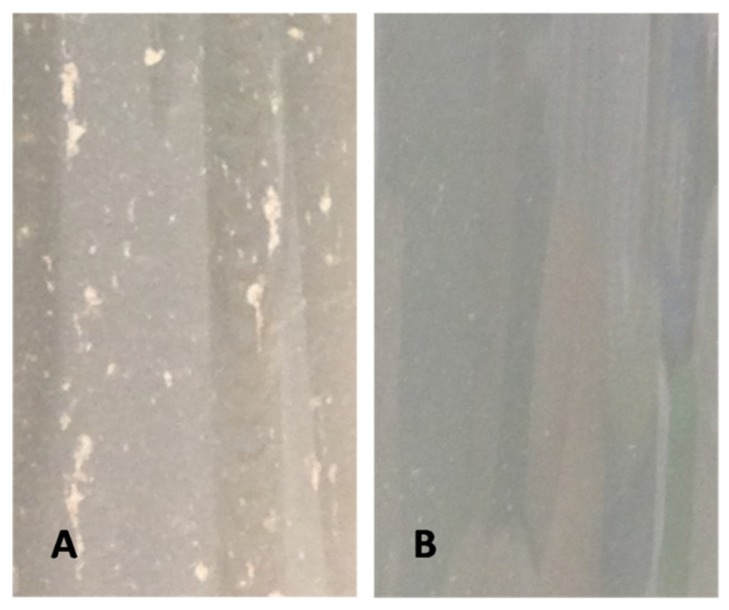
Image of water column with aggregated particles of *Prorocentrum lima* cells and kaolinite clay (**A**) and water column with kaolinite clay alone (**B**).

This rapid clearance corresponded to the cell removal through the flocculation of algal and mineral particles leading to the formation of aggregates, which rapidly settle and further entrain cells during their descent (Figure 5).

**Figure 6:**
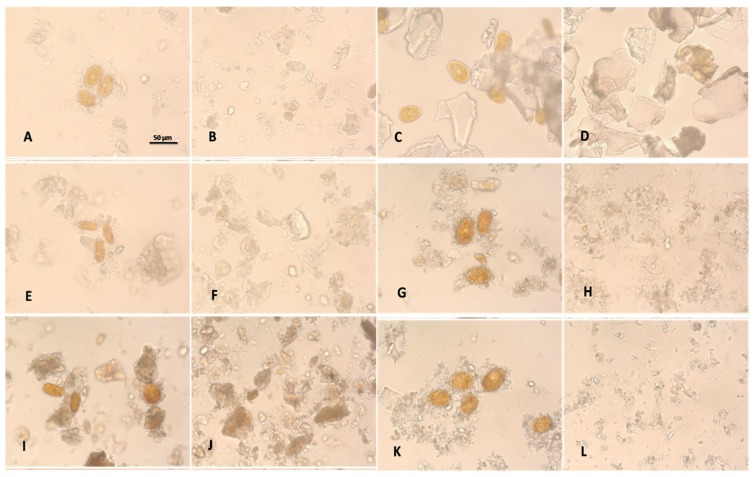
Microscopy images of seawater containing different clays (right column) and flocs formed after incubation with *Prorocentrum lima* (left column)*.* (**A**,**B**) bentonite; (**C**,**D**) bentonite > 45 µm; (**E**,**F**) bentonite Na; **(G**,**H**) Grove Clay; (**I**,**J**) Lendo Clay; and (**K**,**L**) Kaolinite. Scale bar = 50 µm.

Microscopy images showed that clays (especially kaolinite) form aggregates of two or more cells where the mineral particles were bound to the outer membranes of the dinoflagellates (Figure 6).

The removal efficiency (Table 2) seems to be related with composition and particle size of clays. Differences in specific surface area and in surface charges in clays may be responsible for the greater or lesser adsorption with *Prorocentrum lima* cells.

The clays particle size is important. These clays composed of the smallest particles, *i.e.*, bentonite and kaolinite, were the most effective at cell removal, as seen in Table 2 and Figure 6. After the addition of 0.2% of bentonite (80% < 2 µm) and kaolinite (90% < 10 µm), the removal efficiency (RE) of the *Prorocentrum lima* cells was approximately 81.5% in bentonite and 82% in kaolinite within 15 min. The kaolinite Grove clay (69% < 2 µm) and Lendo clay (57% < 2 µm) have similar particle size, which was reflected in an equivalent removal power; both have lower RE than kaolinite. Bentonite Na (80% < 70 µm) and Bentonite > 45 µm were composed by larger particles; therefore, their flocculation power was lower than other studied clays. Bentonite > 45 µm have the highest particle size also the RE reached only 76% after 180 min incubation.

**Table 2:** *Prorocentrum lima* cells removal efficiency (RE) as a function of type of clays and incubation time. Particle size of each type of clay was also included.

Column Content	Time (Minutes)							Clay Size
	*0*	*5*	*15*	*30*	*60*	*120*	*180*	
*P. lima* + bentonite > 45 µm	11.8	33.6	41.6	50.1	72.1	70.7	76	>45 µm
*P. lima* + lendo clay	17.4	35.8	69.5	89.7	96.7	98.8	100	57% < 2µm
*P. lima* + grove clay	15	51.9	77.3	90.4	96.3	100	100	69% < 2 µm
*P. lima* + bentonite	11.7	50.6	81.5	93.7	98.2	99.4	100	80% < 2 µm
*P. lima* + kaolinite	30.1	53.2	82	90.4	97.8	100	100	90% < 10 µm
*P. lima* + bentonite Na	19.8	68.4	74.9	94.2	94.2	99.4	100	80% < 70 µm

The coagulation-flocculation of algae cells using clay is a promising method among the HAB-control treatments and was applied in several coastal waters in Korea, Japan, and China [1]. We developed this environmentally-friendly method using natural clays as effective flocculants for mitigating *Prorocentrum lima* in marine systems. All the clays demonstrated very rapid adsorption to *Prorocentrum lima* within 15 min, which indicates that most adsorption will occur when the dinoflagellates come into contact with the sediment. This is also consistent with results of Wu *et al*., who reported close to 70% adsorption of some microcystins (MC-LR and MC-RR) on natural sediments in 0.5 h (Fubao Bay sediments from Lake Dianchi (Kunming, Yunnan, China) and Meiliang Bay sediments from Lake Taihu (Suzhou, Jiangsu, China) and kaolinite clay were used for the sorption experiments) [25]. In our experiments, despite the differences among the clays, close than 90% removal efficiency of all *Prorocentrum lima* in seawater *versus* the cells with no clays was achieved in 30 min (the exception was bentonite > 45 µm) (Table 2) offering promising results to use these clays for the mitigation of *Prorocentrum lima* blooms.

The adsorption of *Prorocentrum lima* depends on the clay. Results showed that the adsorption capacity of particles decreases substantially with the increase of size. The fine particle-sized fraction had the highest adsorption for *Prorocentrum lima*. Related with that, the removal rate higher than 80% was reached in 15 min with bentonite and kaolinite that have small particle size, at the initial cell concentration of 6.31 × 10^6^ cells/L. It should be considered that the removal rate may be higher with more initial algae concentration. Pan *et al*., in 2006 reported that at the chitosan-modified sepiolite of 11 mg/L the RE after 8 h was 15% at the initial cell concentration of 2 × 10^9^ cells/L and 95% at the cell concentration of 9 × 10^9^ cells/L [19].

According with other studies the adsorption capacity depends mainly on specific surface area of clay and their functional groups such as hydroxyl and carboxyl groups that can act as binding sites for the dinoflagellates [26]. Also the surface charge of dinoflagellates could be increased when cells move from culture mediun to seawater which greatly increased the flocculation potential of the cells [27]. For instance microcystis cells were negatively charged in natural water with pH of 6–10 then bound with organic cations and ions, resulting in aggregation. However, the aggregation effect would not lead to flocculation without a proper gravity function [21]. In our experiments the addition of clay addresses this problem triggering *Prorocentrum lima* sedimentation.

The fast and high efficient removal corresponded to the effective destabilization of algal suspension and fast growth of algal flocs enough to settle fast under static conditions. In our experiments RE after 1 h was higher than 95% with kaolinite, bentonite, Lendo, and Grove clay, however chitosan-modified sepiolite needs 8 h to reach this RE [19]. Sedimentation is regarded as a major challenge for flocculation treatment of dinoflagellate cells [27]. The formation process of flocs was directly confirmed by the flocs structure images (Figure 6). We found that most of the cells that were bound tightly to clays and sedimented in the bottom were alive. The mobile algae escape from the flocs and, therefore, recovery is possible after sedimentation or flocculation. For the field application, the wind-induced currents may cause a serious problem of resuspension. This remains a challenge for future development, where flocculation-capping method may be worth further studies [22].

## 3. Experimental Section

### 3.1. Prorocentrum lima Culture

*Prorocentrum lima* strain CCMP685 (Figure 1) was obtained from the Provasoli-Guillard National Center for Marine Algae and Microbiota (NCMA, East Boothbay, ME, USA) and was grown in 33‰ salinity L1-Si medium prepared in sterile filtered (0.22 µm) natural seawater (75 mg·L^−1^ NaNO_3_, 5 mg·L^−1^ NaH_2_PO_4_·2H_2_O, 4.36 mg·L^−1^ Na_2_·EDTA, 3.15 mg·L^−1^ FeCl_3_·6H_2_O, 0.18 mg·L^−1^ MnCl_2_·4H_2_O, 0.022 mg·L^−1^ ZnSO_4_·7H_2_O, 0.01 mg·L^−1^ CoCl_2_·6H_2_O, 0.0006125 mg·L^−1^ CuSO_4_·5H_2_O, 0.0597 mg·L^−1^ Na_2_MoO_4_·2H_2_O, 0.0013 mg·L^−1^ H_2_SeO_3_, 0.0027 mg·L^−1^ NiSO_4_·4H_2_O, 0.00184 mg·L^−1^ Na_3_VO_4_, 0.00194 mg·L^−1^ K_2_CrO_4_, 0.10 mg·L^−1^ thiamine and 0.005 mg·L^−1^ cyanocobalamine).

The microalgae were cultured at 19 °C under a light intensity of 2000 lux on a 16 h light: 8 h dark photoperiod. Cells were inoculated with an initial concentration of 500 cells/mL into a 2-L Erlenmeyer flask. *Prorocentrum lima* is a benthic dinoflagellate microalga, cells grew forming numerous lumps consisting of dozens of cells that adhered to the flask bottom. In addition, cells secreted extracellular mucus that covered the bottom forming a thin transparent layer. For dinoflagellate count sampling, the cells were gently detached with cell scrapers to prevent cell breakage. The culture was then mildly agitated by hand to uniformly distribute cells in the culture. Afterwards, 1 mL samples were collected and fixed with Lugol’s solution for the initial cell count on a Sedgewick-Rafter counting chamber (Pyser-SGI limited, Edenbridge, UK). This sampling technique was the least invasive of all tested. In fact, the cultures returned to the same original appearance hours later.

Based in the literature the maximum cell concentrations and toxin contents of *Prorocentrum lima* were reached after 35 days of cultivation [19,28]. Therefore, microalgae were cultured for 30 days. Cultured *P. lima* was harvested at the plateau phase and cells were counted using a Sedgewick Rafter counting chamber as described above. Since cell concentration is usually low at lag phase in practical situations, the best timing for clearing up blooms using clays is at the plateau phase around the early senescence phase [19].

### 3.2. Clays Selection

“Caolines de Vimianzo, S.A”, (CAVISA) (Vimianzo, Spain), and “Peloides Termales” (Vigo, Spain), supplied the clays under study. Clays were selected for the specific surface and the particle size.

Figure 2 shows size distribution (granulometry) of the clays. The clay provided by Peloides Termales, “Bentonite Na” was sodium bentonite, and contained particles <70 µm. The studied sample showed a high percentage of phyllosilicates in the total fraction (98%). The clay fraction included the following minerals in decreasing amounts: smectite (56%), sepiolite (29%), and illite (15%) [29,30]. Sodium bentonite expands when wet, absorbing as much as several times its dry mass in water. It was selected because of its excellent colloidal properties.

The clays supplied by CAVISA were bentonite (83% < 2 µm), “Bentonite > 45 µm” (mainly muscovite), kaolinite and kaolinites locally available from the regions Lendo and Grove, near the Galician coast.

### 3.3. Flocculation Assay

Experimental procedure is presented in Figure 3. The cell culture was homogeneous before adding clay. As was described above, after 30 days culture the *Prorocentrum lima* cells were gently detached with cell scrapers to prevent cell breakage. The culture was then mildly agitated by hand to distribute uniformly cells in the culture. Finally, We set up 100 mL water columns with seawater, the corresponding clay (final concentration 0.2% by weight) and the dinoflagellate *Prorocentrum lima*, (from an established *in vitro* culture as it is shown in Figure 3) (Figure 4). The mixtures were allowed to settle by gravity.

1 mL samples at 5 cm depth were taken at the beginning of the experiment and after 5, 15, 30, 60, 120, and 180 min of the clay addition. The cell count of each sample was performed in the Sedgewick Rafter counting chamber with lugol 1:1 (*v*/*v*) as fixative solution. Three independent experiments were performed in duplicate.

### 3.4. Microscopy Images

At the end of the experiment of Figure 3 most of the clay-water mixtures were removed. 10 mL of bottom samples of each water column were taken at the end of the flocculation assay. A small sample of 10 µL was taken from the bottom and was visualized with the microscope. Those samples were prepared at the moment of viewing the state of *Prorocentrum lima* cells after clay treatment (floc images in the Nikon Microscope Eclipse E400 (Nikon corporation, Tokyo, Japan)).

### 3.5. Data Analysis

All experiments were performed in duplicate. Student’s *t*-test for unpaired data, applying a Welch’s correction when necessary, was used for statistical analysis of three independent experiments. In all cases *p* < 0.05 was considered for significance.

## 4. Conclusions

The adsorption behaviors of dinoflagellates to clays need to be investigated more extensively to elucidate the exact adsorption mechanisms. Overall, our findings demonstrate the high potential of natural clay (formed by kaolinite or bentonite) for the adsorption of *Prorocentrum lima* and its importance as a medium that facilitates the removal of dinoflagellates. Understanding the algae flocculation mechanism and designing new environmentally-safe, multi-mechanism methods are important for the HAB control under complex natural conditions. This could help in the design of dinoflagellate monitoring programs and to determine effective strategies for marine water affected by severe HABs related to diarrheic shellfish toxins.
